# Reduced Volume ‘Daily Max’ Training Compared to Higher Volume Periodized Training in Powerlifters Preparing for Competition—A Pilot Study

**DOI:** 10.3390/sports6030086

**Published:** 2018-08-29

**Authors:** Patroklos Androulakis-Korakakis, James P. Fisher, Panagiotis Kolokotronis, Paulo Gentil, James Steele

**Affiliations:** 1 School of Sport, Health, and Social Science, Southampton Solent University, Southampton SO14 0AA, UK; james.fisher@solent.ac.uk (J.P.F.); james.steele@solent.ac.uk (J.S.); 2 AEK Athens Powerlifting, 14341 Athens, Greece; pkolokotronis@gmail.com; 3 Faculty of Physical Education, Federal University of Goiás, Goiania 74690-900, Brazil; paulogentil@hotmail.com; 4 Ukactive Research Institute, London WC1R 4HE, UK

**Keywords:** powerlifting, daily max, training volume, muscle strength, resistance training

## Abstract

The present study looked to examine reduced volume ‘daily max’ (near max loads) training compared to higher volume periodized training in powerlifters preparing for competition. Ten competitive powerlifters were split into 2 groups (MAX group and PER group) and participated in a 10-week training intervention either following a “daily max” training protocol or a traditional periodized training protocol while preparing for competition. All participants underwent 1RM testing for squat (SQ), bench press (BP) and deadlift (DL) prior to the 10-week intervention. The MAX group performed single sets of single repetitions using a load equating to an RPE rating of 9–9.5 while the PER group performed higher volume periodized training with loads ranging from 70%1RM up to 93%1RM as well as a taper at the final weeks of the training intervention. Both groups were tested after the 10-week training intervention at the Greek IPF-affiliate National Championships. In the PER group, powerlifting (PL) total increased for P1 and P3 by 2% and 6.5% respectively while P2 experienced no change. In the MAX group PL total increased for P1 and P2 by 4.8% and 4.2% respectively while it decreased by 0.5%, 3.4% and 5% for P3, P4 and P5 respectively. In the MAX group peri PL total increased for P1–4 by 3.6%, 4.2%, 4.5% and 1.8% respectively while it decreased by 1.2% for P5. The results of this pilot study show that single-set, single-rep, RPE based ‘daily max’ training may be a favorable strategy for some beginner-intermediate powerlifters preparing for competition while it may lead to performance decreases for others. Further, it suggests that performance may be comparable to traditional periodized training during shorter training cycles, though future work with larger samples is needed to further test this. Practically ‘daily max’ training may be useful for PL athletes looking to maintain strength during periods with limited training time available.

## 1. Introduction

Powerlifting (PL) is a strength sport based on the 3 powerlifts; the squat (SQ), the bench press (BP) and the deadlift (DL). In competition, a powerlifter has 3 single-repetition attempts in each of the powerlifts with the goal of achieving the highest PL total possible [1]. The PL total is calculated by adding the highest successful attempt in each of the 3 powerlifts. PL has existed as a strength sport for a few decades, but has started to receive more mainstream attention in the last 10–15 years. As PL performance started receiving more attention by the strength and conditioning community, so to have the training methods to improve such performance. Most of the available resources on PL training make use of the existing literature on resistance training approaches for improving strength but the actual studies investigating the effects of different training approaches on PL performance in powerlifters are very limited. However, powerlifters worldwide have successfully utilized a variety of approaches in preparation for competitions and, despite the considerable variation, many have achieved world-record performances. These approaches can range from those utilizing a high-volume and submaximal loads, to those using a low-volume and near maximal loads. Most resources used to support training for PL have scientific underpinning from up to date research on resistance training in general. Yet the literature looking directly at PL athletes and their training methods is currently very limited.

A common approach to PL competition preparation is the use of the traditional model of periodization where the athlete begins with a preparatory period consisting of high volume training with loads ranging from 70–85%1RM [2]. This is then followed by a gradual reduction in training volume and a gradual increase in training load, moving from the range of 70–85%1RM to a heavier 80–97.5%1RM as the competition approaches [3]. Regardless of the periodization model used by a PL athlete or coach, the vast majority of powerlifters will incorporate both high and low volumes of training as well as high and low training loads when preparing for a competition.

A contrasting method of training that has been utilized by Olympic weightlifting coaches and athletes for many years involves frequent training with very high loads, usually above 85%1RM, but with a very low training volume often using sets of single repetitions. Zourdos et al. [4] examined the effect of performing the back SQ daily with very heavy loads (a 1RM followed by 5 sets of 3 repetitions at 85%1RM or 2 repetitions at 90%1RM) and found that 1RM strength improved over a period of 37 days. Training with low volume but with very high loads may be most appropriate for PL performance as it addresses the element of specificity; which in this case is a high load single repetition. In essence, practicing the demands of the sport of PL (i.e., 1RMs) may be effective in increasing performance, at least during short training cycles. However, some PL coaches and athletes still avoid very high loads for as long as possible as it is thought that greater central fatigue that occurs under higher loads [5] though recent work suggests central fatigue is similar irrespective of load [6]. Many still consider regular heavy load training to be undesirable and that such training can often lead to injury due to the technical breakdown that occurs for some powerlifters when approaching the 1RM [7]. The incorporation of very high loads during a powerlifter’s training cycle is highly debated; some coaches advocate the frequent use of loads above 90%1RM while other coaches argue against it and promote high volume training with loads around 70–85%1RM. Despite the different opinions that exist on the subject by coaches and athletes, the available data so far suggests that frequent high load training may be beneficial for in terms of increasing strength [8,9]. There is a relative lack of research comparing “daily max” training with any other approach particularly in powerlifters who, it might be speculated, may have the most to gain from its application along with weightlifters. Thus the aim of the present pilot study was to compare the implementation of a ‘daily max’ style training approach to that of a traditional periodized training approach in PL athletes preparing for competition over a 10-week training cycle.

## 2. Materials and Methods

### 2.1. Experimental Approach to the Problem

Using a quasi-experimental study design, a traditional periodized training program was compared to a very low, high load ‘daily max’ program in Greek competitive powerlifters. Each training program was used in preparation for the Hellenic Powerlifting Federation (HPF) national championships in Athens, Greece. HPF is the Greek International Powerlifting Federation (IPF) affiliate. Each training program was performed over a training period covering a 10-week cycle with pre testing of 1RM performed at baseline and post testing including actual competition performances. The total length of the study, including testing weeks was 11 weeks.

### 2.2. Participants

Approval by the relevant ethics committee at the researchers’ institution was initially obtained (Health, Exercise, and Sport Science Ethics Committee ID No. 890). Following this, a total of 10 males (age = 27 ± 6 years, body mass = 90.5 ± 16.7 kg, height = 178.9 ± 6.2 cm) from the team AEK PL, all with at least 1 year of PL experience and at least 2 years of resistance training experience, were recruited. Prior to the training intervention, participants were following a PL program that incorporated a moderate amount of training volume as well as both moderate and high loads (70–90%1RM). Sample size was justified based upon the availability of PL athletes and not based upon any a priori statistical criteria. The level of the powerlifters that took part in the study ranged from beginner to intermediate with 2 ± 1 years of PL experience, and 5 ± 2 years of resistance training experience. PL level was established using the latest IPF classification standards. All participants provided written informed consent prior to participation and were thoroughly provided with specific instructions pertaining to the study procedures. All participants were asked to refrain from any other form of exercise, especially resistance training, as it could interfere with their performance during training sessions. Participants that were unable to attend certain training sessions were required to inform the head coach of the PL team in advance in order to keep training records updated. Any participants that missed a supervised training session were required to complete the training session on their own and provide video footage of all their working sets to the head coach.

### 2.3. Group Assignment

The participants were assigned to either the MAX group (*n* = 5, heavy load, very low volume, “daily max” training) or the PER group (*n* = 5, traditional periodized PL training program). The group assignment process was non-randomized. Instead, in a process managed by the team’s head coach, participants were paired based upon current performance and then divided evenly between the two groups.

### 2.4. Testing

All participants underwent 1RM testing prior to the 10-week training intervention. The initial 1RM testing was performed in a competition-like setting, requiring participants to test their SQ, BP and DL 1RM all on the same day with 3 attempts allowed for each powerlift. Post training intervention, participants tested their 1RM at the HPF National Championships. As per the rules of the IPF, all participants were allowed three 1-repetition attempts for each powerlift. Warm-ups took place in the designated warm-up area at the competition venue, where 20–30 min had been allocated to the athletes in order to prepare for their maximal attempts. Participants in both groups performed the same warm-up procedure, gradually increasing load and decreasing repetitions as they approached a load approximately 10% lighter than their first attempt. Depending on the competitors in one’s category, waiting time between attempts ranged from 3–5 min. Between each powerlift event, athletes had the opportunity to rest for approximately 15–20 min before beginning to warm-up for the next event. Upon completion of the training interventions and post testing, competition athletes were also asked to complete a questionnaire regarding their training protocol with questions about its effect on: enjoyment, adherence, effectiveness and impact on injuries. The questionnaire was based on a 5-point likert scale and responses above the 3 points mark on the Likert scale implied a positive response.

### 2.5. Training

Prior to the first testing session all participants first underwent 4 weeks of familiarization with the resistance training version of the RPE scale based upon ‘repetitions in reserve’ [10,11]. The athletes were required to implement the modified RPE scale in their current training. This was overseen and supervised by the coach who observed 90% of the training sessions for both groups. After the 4-week familiarization period the two groups began their assigned training intervention (MAX or PER), which was completed over a 10-week period. Most training sessions were completed at the Olympic Weightlifting training hall in the Athens Olympic Complex.

The training protocol followed by the PER group consisted of a 4-week preparatory mesocycle where training load was kept around 70–80%1RM. It was then followed by a 4-week transitional mesocycle where training volume remained high and training load slightly increased to 75–85%1RM. The PER group training protocol ended with a 2-week peaking block were volume decreased and training load reached its highest values (90–93%1RM). The PER group trained 3 times per week and performed the powerlifts with the following frequency: SQ on day 1 and day 3, BP on all 3 days and DL on day 2. Athletes in the PER group performed multiple working sets during every training session. No accessory exercises were performed by the PER group. The RPE value for all working sets was also recorded. Athletes were instructed to not surpass RPE 9 on their working sets. Training details for the PER group can be found in Table 1. The complete training protocol is available as a supplementary material.

The training protocol followed by the MAX group consisted of 1 set of a single repetition at RPE 9–9.5 (i.e., where another repetition could not be performed if attempted, though a slight increase in load might be possible) for 3 training sessions per week. The modified rating of perceived effort (RPE) scale [1] for self-determining load, allows athletes and coaches to become more flexible when designing training programs. Instead of prescribing a specific load based on the athletes’ 1RM, coaches will often prescribe a specific RPE number that the athlete must reach for a selected amount of sets and repetitions (eg: 3 × 3 @8RPE). This allows the athlete to self-select the most appropriate load based on preparedness. The MAX group performed the powerlifts with the same frequency as the PER group. The MAX group performed the SQ on day 1 and day 3, the BP on all 3 days and the DL on day 2. The MAX group decreased its training sessions to 2 on the week of the competition for recovery purposes, performing the SQ and BP on 2 sessions and the DL on 1. Similarly, to the PER group, the MAX group performed no accessory exercises. Training details for the MAX group can be found in Table 2. The full training protocol is available as a supplementary material.

### 2.6. Data Analysis

As this was a pilot study with a small sample size, inferential statistics were not applicable and instead individual participant responses are presented. Absolute and percentage change was calculated for pre and post SQ, BP, DL 1RM and PL total for the PER group. Absolute and percentage change was calculated for pre, peri and post SQ, BP, DL 1RM and PL total for the MAX group. Participant and training characteristics were analyzed using descriptive statistics. Analysis was performed using JASP (version 0.8.6, University of Amsterdam, Amsterdam, The Netherlands).

## 3. Results

Two participants from the PER group suffered minor injuries, one related to the training protocol and the other one unrelated, and were excluded from the rest of the training intervention and therefore not included in the data analysis. The remaining 8 participants were all included in the data analysis as they successfully completed the training intervention. Training characteristics for both groups can be found in Table 3.

In the PER group, participant 1 (P1) and participant 3 (P3) increased their PL total by 2% and 6.5% respectively while participant 2 (P2) maintained his pre PL total. In the MAX group, participant 1 (P1), participant 2 (P2) and participant 4 (P4) increased their PL total by 4.8%, 4.2% and 3.4% respectively while participant 3 (P3) and participant 5 (P5) decreased their PL total by 0.5% and 5% respectively. When looking at the pre-peri PL total differences in the MAX group, P1, P2, P3 and P4 increased their PL total by 3.6%, 4.2%, 4.5% and 1.8% respectively while P5 decreased his total by 1.2%.

In the PER group, post SQ 1RM increased for P1 and P2 by 2.5% and 2.8% respectively. In the MAX group, P1 and P3 increased their post SQ 1RM by 6.6% and 3.3% respectively while P2, P4 and P5 experienced no change in their SQ 1RM. P1, P2 and P4 increased their peri SQ 1RM by 6.6%, 3.5% and 1.1% respectively while P3 and P5 experienced no change. In the PER group, post BP 1RM increased for P1 by 1.7% while there was no change for P2 and P3. In the MAX group, P1 experienced no change in BP performance while P2 increased post BP 1RM by 3.6%. P3, P4 and P5 decreased their post BP 1RM by 6.4%, 12% and 11.4% respectively. P2 and P4 increased their peri BP 1RM by 5.4% and 1.8% respectively while P1, P3 and P5 experienced no change in peri BP 1RM. In the PER group post DL 1RM increased for P1 and P3 by 2.1% and 14% respectively while P2 did not experience any change. In the MAX group, post DL 1RM increased for P1 and P2 by 6% and 8.3% respectively. P3 did not experience any change and P4 and P5 experienced a 2.1% and 6.6% decrease in post DL 1RM. Peri DL 1RM increased for P1, P2, P3 and P4 by 9%, 4.2%, 11.7% and 2.4% respectively. Peri DL 1RM decreased by 3.2% for P5.

Participants 1–5 in the MAX group achieved their peri SQ 1RM in the following weeks: 8, 9, 7, 6 and 5. Peri BP 1RM was achieved during weeks: 1, 4, 4, 3 and 3. Peri SQ 1RM was achieved during weeks: 9, 6, 4, 5 and 7. During the 10-week training intervention, there were 3 failed “daily max” attempts in the MAX group (2 in the BP and 1 in the DL). All individual powerlifting characteristics can be found on Table 4 for the PER group and on Table 5 for the MAX group.

## 4. Discussion

The present pilot study examined the effect of 10 weeks of “daily max” style training compared to a traditional higher volume varying load approach in PL athletes preparing for competition. 2 out of 3 participants in the PER group increased their PL total by 2% and 6.5% while 2 out of 5 participants in the MAX group increased their PL total by 4.8%, and 4.2% and 3 decreased their total by 0.5%, 3.4% and 5%. When looking at the peri PL total for the MAX group, 4 participants increased their PL total by 3.6%, 4.2%, 4.5% and 1.8% while 1 participant decreased his PL total by 3.2%. Since competition performance decreased in 3 out of 5 participants of the MAX group “daily max” training may only be appropriate for certain athletes when preparing for competition while a traditional periodized training may be a more appropriate competition preparation approach. The results of this pilot study also imply that there may be a minimum effective training dose when trying to increase 1RM strength in strength athletes as well as well-trained participants.

The individual data demonstrates that “daily max” training may be more effective for shorter training cycles (4–7 weeks) as most participants in the MAX group experienced higher 1RM increases during peri-training rather than post. 3 participants in the MAX group lifted the heaviest loads for SQ and DL during weeks 4–7 and 2 participants on weeks 8 and 9. 4 participants in the MAX group lifted the heaviest loads during weeks 3–4 for the BP while 1 participant lifted the heaviest loads on week 1. The weeks where the MAX group achieved its highest SQ and DL numbers imply that a 4–7 weeks training cycle may have been more effective when implementing a “daily max” training approach, at least for the SQ and DL. Besides implying that a “daily max” protocol may be better utilized during shorter training cycles, the above data demonstrate how different peaking may be for each individual athlete. Participants in the MAX group achieved their best SQ and DL performances all on different weeks, implying that planning a peaking cycle may be more complicated and individual than previously thought. The above data also demonstrate that the BP may be more training-volume dependent than the SQ and DL as the participants of the MAX group achieved their greatest performance earlier than the SQ and DL. These findings contradict some of the previous literature that has found the lower body strength to be more training volume dependent than upper body strength [12,13]. Similarly to the SQ findings of this study, Zourdos et al. [4] found that squatting with low volume but very high loads consecutively for 37 days (i.e., around 5 weeks) increased SQ 1RM in 2 powerlifters and a weightlifter.

The peri results of the MAX group may have been a result of the increased skill acquisition component of “daily max” training. The fact that the participants of the MAX group performed only single-set, single-repetition sets with high loads throughout the entire training intervention might have allowed them to become better at performing that specific task as seen in previous research looking at repeated 1RM tests as a mean of increasing strength [9]. Literature supports the idea that increases in strength can be achieved with both high and low volumes of training [14,15,16] but there is currently very little data on powerlifters and specifically the ‘daily max’ method. Zourdos et al. [4], is the only study that looked at something similar to a ‘daily max’ protocol but as previously mentioned it investigated only the SQ and its participants trained with a very high training frequency. High frequency training has been shown to be effective in increasing strength in weightlifters, powerfliters, and trained subjects and is one method to increase overall training volume [4,17]. However, previous studies have shown that strength likely depends more on load than training volume [8,9], something that may explain the MAX group’s peri results, even though the PER group also trained with loads that would be considered heavy (85–90%1RM). Indeed, as previously noted, Mattocks et al. [9] compared higher volume training with repeated performances of 1RMs in untrained participants finding similar strength gains for 1RM chest press and knee extension in the group training with repeated 1RMs. This group also performed a considerably lower overall training volume. The results of the present study support the idea that strength increases with very low volumes of training may be possible in trained subjects as long as training load is kept very high, despite moderate training frequency. 

An interesting difference between the 2 groups is that the PER group had a 2-week peaking phase planned prior to competition. Training volume decreased while training load increased (though did not reach the same relative loads as the MAX group), allowing for the PER group to maximize performance on the day of the competition. Similar peaking approaches are utilized by PL athletes of all levels as a mean of optimizing performance on the day of the competition [18]. In contrast, the MAX group continued performing the same training throughout the study and simply reduced frequency to 2 days for the final week and rested for 5–7 days prior to the competition. Similarly, when investigating the effects and mechanisms of tapering in maximizing muscular strength, Pritchard et al. [3] concluded that a training cessation of 2–4 days may be optimal and all that is required for enhanced maximal muscular strength, and that less than a week of training cessation may be optimal for performance maintenance. This may explain why the MAX group’s performance peri-training was higher than the group’s competition performance as well as imply that additional days of absolute rest may have increased the group’s competition performance. As noted though it is not clear if similar strength gains might have occurred in the PER group over this time period as we did not collect peri-training max strength data.

Since there were only 3 failed “daily max” attempts among the 5 participants of the MAX group throughout the 10-week training intervention, the results of this pilot study suggest that PL athletes may be able to effectively self-adjust load by using the modified RPE scale and that doing so may be a beneficial approach to load management in PL training [10,19]. The PER group followed a percentage 1RM based training plan, though were instructed to not exceed RPE 9, while the MAX group self-adjusted load until an RPE of 9–9.5 was reached. The participants of the MAX group largely managed to select appropriate loads and successfully progressed, managing higher loads than they could handle prior to the study without failure or experiencing injury. Only in the DL were participants seemingly less able to do so with an average RPE of 8.9. The RPE familiarization period prior to the main training sessions could have positively contributed to the PL athletes’ successful utilization of the modified RPE as some of them were not familiar with the scale.

In the present study the MAX group performed less than 10% of the overall volume-load that the PER group performed in all powerlifts despite training every powerlift with the same frequency. Despite the lack of training volume, 2 of the 5 MAX group’s participants were able to increase their competition performance, while 4 of the MAX group’s participants experienced strength increases peri-training. The differences in training volume may also explain the performance decrease that the 3 participants of the MAX group experienced in competition as there were no performance decreases in the PER group. Even though the MAX group’s participants constantly addressed the element of specificity by performing single sets of single repetitions with heavy loads over 10 weeks, only 2 out of 5 participants increased their competition performance. The decrease in competition performance seen in the MAX group demonstrates that only certain PL athletes may be able to maintain, or perhaps increase strength with minimal volume for periods longer than 10 weeks.

When looking at the participants’ questionnaire data in Table 6, all responses were above the 3 points mark on the Likert scale implying positive responses to all questions. Participants in both groups found their training protocols enjoyable, easy to adhere to and the workload manageable. Buckner et al. [20] found that participants enjoyed 1RM-testing based training more than higher repetition training, which may possibly explain the responses of the participants of the MAX group. It is worth mentioning that on the question “How effective was your training protocol on optimizing performance for competition day?”, participants of the PER group reported over a 1 point difference higher than the MAX group, indicating that the traditional periodized program was viewed as more appropriate for competition preparation.

The limitations of the study must be noted. The small sample size in combination with the level of the participants (beginner–intermediate), limits the applications that the results of the study have. It should be noted though that the 10 participants originally recruited represented the entire AEK PL team. Recruitment of participants from this type of population is typically difficult and even more so for training intervention studies but it is possible, as shown in the present study. Future studies may need to engage multiple PL teams in order to achieve sufficient samples for adequate power in statistical comparisons. The differences in load used by the 2 groups is also a limitation. The MAX group constantly trained with loads above 90%1RM while the PER group only utilized loads of 90%1RM and above during the final weeks of the study since it had a 2-week peaking phase, which may have placed the PER group at disadvantage. Another limitation is that the post testing was an actual competition. Though, this could be considered a strength as it meant that ecologically valid performance was the outcome. However, participants’ competition performance could have been affected by poor attempt selection on competition day. The participants were tested during a national-level PL competition, which may have led to unrealistic 3rd attempts in hopes of attaining a podium placement. The final results of the participants could have been different had they been more conservative in their attempt selection. “Daily max” training may not be an effective year-round approach for powerlifters and strength athletes as further research is needed in order to fully understand its place and use in ones’ training.

## 5. Conclusions

In conclusion, the results of this study suggest that single-set, single-rep RPE based “daily max” training may be an effective short-term training approach for beginner-intermediate PL athletes as well as PL athletes looking to maintain strength during periods with limited training time available. Traditional periodized training may be more effective in preparing PL athletes for competition and “daily max” training should be utilized with caution as it may lead to deleterious effects on PL performance. 

## Figures and Tables

**Table 1:** PER Group Training Session Ranges.

Session Ranges	Week 1	Week 2	Week 3	Week 4	Week 5	Week 6	Week 7	Week 8	Week 9	Week 10
**SQ**										
Working set range	4–6	7–8	6–7	7–8	5–6	5–6	4–5	6–7	4–5	3–5
Repetition range	2–3	2–3	2–3	2–5	2–3	2–3	2–3	1–3	2–3	1–2
%1RM range	70–85%	70–85%	70–85%	75–85%	70–85%	70–80%	70–85%	70–90%	70–90%	70–93%
Sessions	2	2	2	2	2	2	2	2	2	2
**BP**										
Working set range	6–15	7–8	7–9	7–8	6–7	7–11	8–10	7–9	3–5	3–5
Repetition range	3–4	3–4	1–4	2–3	2–3	1–7	2–3	2–3	2–3	1–3
%1RM range	70–85%	70–85%	70–90%	70–85%	70–85%	55–90%	70–85%	70–85%	70–90%	70–93%
Sessions	3	3	3	3	3	3	3	3	3	2
**DL**										
Sets	6	8	9	8	6	5	6	6	5	3
Repetitions range	2–3	2–3	1–3	2–3	3	2–3	2–3	3	1–2	1
%1RM range	70–85%	70–80%	70–85%	70–85%	70–80%	70–80%	70–90%	70–80%	70–90%	70%
Sessions	1	1	1	1	1	1	1	1	1	1

**Table 2:** MAX Group Training Session Ranges.

Session Ranges	Week 1	Week 2	Week 3	Week 4	Week 5	Week 6	Week 7	Week 8	Week 9	Week 10
**SQ**										
Working set range	1	1	1	1	1	1	1	1	1	1
Repetition range	1	1	1	1	1	1	1	1	1	1
RPE range	9–9.5	9–9.5	9–9.5	9–9.5	9–9.5	9–9.5	9–9.5	9–9.5	9–9.5	9–9.5
Sessions	2	2	2	2	2	2	2	2	2	2
**BP**										
Working set range	1	1	1	1	1	1	1	1	1	1
Repetition range	1	1	1	1	1	1	1	1	1	1
RPE range	9–9.5	9–9.5	9–9.5	9–9.5	9–9.5	9–9.5	9–9.5	9–9.5	9–9.5	9–9.5
Sessions	3	3	3	3	3	3	3	3	3	2
**DL**										
Sets	1	1	1	1	1	1	1	1	1	1
Repetitions	1	1	1	1	1	1	1	1	1	1
RPE	9–9.5	9–9.5	9–9.5	9–9.5	9–9.5	9–9.5	9–9.5	9–9.5	9–9.5	9–9.5
Sessions	1	1	1	1	1	1	1	1	1	2

**Table 3:** Training Characteristics.

Training Outcome	MAX (*n* = 5)	PER (*n* = 3)
Total training sessions	26.4 ± 2.0	30 ± 0
**SQ**		
Total sessions	18 ± 0.8	18.3 ± 2.8
Total volume (kg)	3138 ± 612	37,609 ± 6561
Average RPE	9.1 ± 0.10	8.6 ± 0.08
**BP**		
Total sessions	25.8 ± 2.5	30 ± 0
Total volume (kg)	3002 ± 609.4	55,655.6 ± 9897.8
Average RPE	9.2 ± 0.155	8.4 ± 0.254
**DL**		
Total sessions	9.6 ± 0.5	10 ± 0
Total volume (kg)	1790 ± 373	19,433 ± 2646
Average RPE	8.9 ± 0.4	8.4 ± 0.1

Note: Results are mean ± SD.

**Table 4:** Individual Powerlifting Characteristics (PER group).

Characteristic	Participant 1	Participant 2	Participant 3
**SQ**			
Pre 1RM (kg)	200	175	175
Post 1RM (kg)	205	175	180
Pre-Post 1RM Δ (kg)	5	0	5
Pre-Post 1RM Δ (%)	2.5	0	2.8
**BP**			
Pre 1RM (kg)	145	120	140
Post 1RM (kg)	147.5	120	140
Pre-Post 1RM Δ (kg)	2.5	0	0
Pre-Post 1RM Δ (%)	1.7	0	0
**DL**			
Pre 1RM (kg)	230	165	200
Post 1RM (kg)	235	165	230
Pre-Post 1RM Δ (kg)	5	0	30
Pre-Post 1RM Δ (%)	2.1	0	14
**Total**			
Pre (kg)	575	460	515
Post (kg)	587	460	550
Pre-Post 1RM Δ (kg)	12.5	0	35
Pre-Post 1RM Δ (%)	2	0	6.5

**Table 5:** Individual Powerlifting Characteristics (MAX group).

Characteristic	Participant 1	Participant 2	Participant 3	Participant 4	Participant 5
**SQ**					
Pre 1RM (kg)	145	210	145	215	155
Peri 1RM (kg)	155	217.5	145	217.5	155
Post 1RM (kg)	155	210	150	215	155
Pre-Post 1RM Δ (kg)	10	0	5	0	0
Pre-Post 1RM Δ (%)	6.6	0	3.3	0	0
Pre-Peri 1RM Δ (kg)	10	7.5	0	2.5	0
Pre-Peri 1RM Δ (%)	6.6	3.5	0	1.1	0
Week of Peri 1RM	8	9	7	6	5
**BP**					
Pre 1RM (kg)	100	135	120	132.5	92.5
Peri 1RM (kg)	100	142.5	120	135	92.5
Post 1RM (kg)	100	140	112.5	117.5	82.5
Pre-Post 1RM Δ (kg)	0	5	−7.5	−15	−10
Pre-Post 1RM Δ (%)	0	3.6	−6.4	−12	−11.4
Pre-Peri 1RM Δ (kg)	0	7.5	0	2.5	0
Pre Peri 1RM Δ (%)	0	5.4	0	1.8	0
Week of Peri 1RM	1	4	4	3	3
**DL**					
Pre 1RM (kg)	160	230	160	240	155
Peri 1RM (kg)	175	240	180	246	150
Post 1RM (kg)	170	250	160	235	145
Pre-Post 1RM Δ (kg)	10	20	0	−5	−10
Pre-Post 1RM Δ (%)	6	8.3	0	−2.1	−6.6
Pre-Peri 1RM Δ (kg)	15	10	20	6	−5
Pre-Peri 1RM Δ (%)	9	4.2	11.7	2.4	−3.2
Week of Peri 1RM	9	6	4	5	7
**Total**					
Pre (kg)	405	575	425	587.5	402.5
Peri (kg)	420	600	445	598.5	397.5
Post (kg)	425	600	422.5	567.5	382.5
Pre-Post Δ (kg)	20	25	−2.5	−20	−20
Pre-Post Δ (%)	4.8	4.2	−0.5	−3.4	−5
Pre-Peri Δ (kg)	15	25	20	11	−5
Pre-Peri Δ (%)	3.6	4.2	4.5	1.8	−1.2

**Table 6:** Questionnaire responses.

Question	MAX (*n* = 5)	PER (*n* = 3)
How enjoyable was the training protocol that you were assigned to?	3.8 ± 0.4	3.6 ± 1.5
How easy was it to adhere to your training protocol?	3.4 ± 1.3	3.6 ± 0.5
How manageable was the workload of your training sessions?	3.6 ± 0.5	3.3 ± 0.5
How effective was your training protocol in covering your training needs?	3.2 ± 0.8	3.6 ± 1.1
How likely are you to use the same or a similar training protocol in the future?	3 ± 0.7	3.6 ± 1.5
How much did the training protocol impact current or past injuries?	1	1.3 ± 0.57
How effective was the training protocol on optimising performance for competition day?	2.8 ± 0.4	4.3 ± 0.5
How much did exogenous factors (stress, lack of sleep) affect your performance on competition day?	3.8 ± 1.6	2.6 ± 1.1
How effective was the coach’s involvement in improving performance during the training sessions?	4.2 ± 1	3.6 ± 0.5
How accurately did you follow the protocol’s guidelines (eg: RPE or load/reps assigned)?	4	3 ± 1.7
How helpful was the RPE familiarisation period? (the weeks prior to the study)	3.8 ± 1	3.3 ± 0.5
How confident were you at utilising the RPE scale during training sessions?	3.6 ± 0.5	3.6 ± 1.5

Note: Results are mean ± SD.

## Supplementary Materials

The following are available online at http://www.mdpi.com/2075-4663/6/3/86/s1.
